# Sonic Hedgehog-Signalling Patterns the Developing Chicken Comb as Revealed by Exploration of the *Pea-comb* Mutation

**DOI:** 10.1371/journal.pone.0050890

**Published:** 2012-12-05

**Authors:** Henrik Boije, Mohammad Harun-Or-Rashid, Yu-Jen Lee, Freyja Imsland, Nicolas Bruneau, Agathe Vieaud, David Gourichon, Michèle Tixier-Boichard, Bertrand Bed’hom, Leif Andersson, Finn Hallböök

**Affiliations:** 1 Department of Neuroscience, Uppsala University, Uppsala, Sweden; 2 Science for Life Laboratory, Department of Medical Biochemistry and Microbiology, Uppsala University, Uppsala, Sweden; 3 Institut National de la Recherche Agronomique, AgroParisTech, UMR1313 Animal Genetics and Integrative Biology, Jouy-en-Josas, France; 4 Institut National de la Recherche Agronomique, UE1295 Poultry Experimental Platform of Tours, Nouzilly, France; 5 Department of Animal Breeding and Genetics, Swedish University of Agricultural Sciences, Uppsala, Sweden; University of Oxford, United Kingdom

## Abstract

The genetic basis and mechanisms behind the morphological variation observed throughout the animal kingdom is still relatively unknown. In the present work we have focused on the establishment of the chicken comb-morphology by exploring the *Pea-comb* mutant. The wild-type single-comb is reduced in size and distorted in the *Pea-comb* mutant. Pea-comb is formed by a lateral expansion of the central comb anlage into three ridges and is caused by a mutation in *SOX5*, which induces ectopic expression of the SOX5 transcription factor in mesenchyme under the developing comb. Analysis of differential gene expression identified decreased Sonic hedgehog (SHH) receptor expression in Pea-comb mesenchyme. By experimentally blocking SHH with cyclopamine, the wild-type single-comb was transformed into a Pea-comb-like phenotype. The results show that the patterning of the chicken comb is under the control of SHH and suggest that ectopic SOX5 expression in the Pea-comb change the response of mesenchyme to SHH signalling with altered comb morphogenesis as a result. A role for the mesenchyme during comb morphogenesis is further supported by the recent finding that another comb-mutant (*Rose-comb*), is caused by ectopic expression of a transcription factor in comb mesenchyme. The present study does not only give knowledge about how the chicken comb is formed, it also adds to our understanding how mutations or genetic polymorphisms may contribute to inherited variations in the human face.

## Introduction

**Figure 1:**
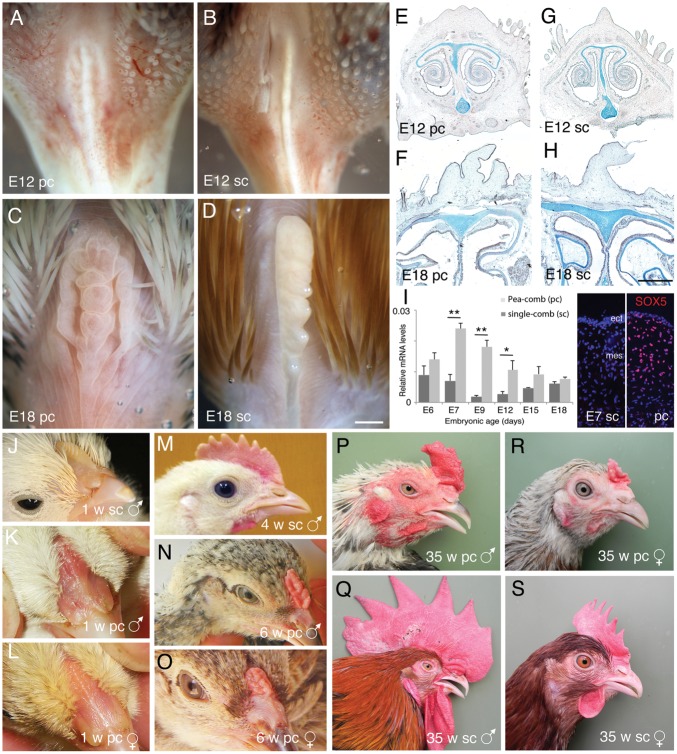
Comparison of Pea- and single-comb with respect to comb morphology, Alcian blue cartilage staining and SOX5 expression. (A–D) Morphology of E12 and E18, Pea- and single-combs. (E–H) Alcian blue stained cross section of E12 and E18 Pea- and single-combs to visualize cartilaginous structures. Note that in spite of the differences in Pea- and single-comb morphology the underlying cartilage structures are normal. The sections are not exactly on the same level. (I) Bar graph with qRT-PCR results and fluorescence micrographs of immunohistological analysis of SOX5 mRNA levels. Bar graph data are normalized to the ß-actin mRNA levels and is related to the ß-actin mRNA level. Bars±s.e.m., ANOVA, n>4 combs per sample * p<0.05, **p<0.005. (J–S) Photographs of Pea- (K, L, N–Q) and single combed (J, M, R, S) chicken of the sex and ages as indicated in the figure. E; embryonic day, pc; Pea-comb, sc; single-comb, w; weeks. Scale bars are 250 µm.

To understand the mechanisms behind morphogenesis and to gain insights into the genetic basis of the great morphological variation observed throughout the animal kingdom has been one of the principal objectives in biology. Morphological variation has unequivocally been important during speciation but also when it comes to marking individuality within a population in a social context. Subtle variations in face morphology are in this context salient but our knowledge how such variations are generated, both from a mechanistic and genetic perspective, is still relatively poor. Pea-comb was one of the chicken comb-variants used by William Bateson in 1902 as traits to demonstrate the first examples of Mendelian inheritance in animals [1]. The wild-type comb with its single central blade often denoted single-comb, is distorted by the dominant *Pea-comb* (*P*) allele. The Pea-comb is initially formed by a lateral expansion of the single comb anlage into three rows of papillae; it expands laterally with an increasingly irregular shape and become reduced in size (Fig. 1 A–D, J–S). The chicken comb originates from a region on the upper beak, posterior to the fronto-nasal process and is first visible as a narrow midline ridge, the comb-ridge, at embryonic day 6–7 (E6–7) [2]. The single-comb has one row of papillae that are formed from local mesenchyme condensations along the initial comb-ridge and they present the beginnings of the comb serrations, which are characteristics of the adult single-comb and which are distorted in the Pea-comb. Further growth of the comb involves local proliferation of ectoderm and comb mesenchyme with structural rearrangements generating a loose fibrous central connective tissue consisting of extracellular matrix covered by dermis and epidermis [3]. Heterotopic transplantation of embryonic comb-ridge ectoderm and mesenchyme performed during the 60′s implied that control of the lateral and longitudinal morphology of the single-comb phenotype is exerted by epithelial-mesenchymal interactions starting at E4–5 [4], [5]. The comb is formed right after the major facial prominences are laid out, which establish the fundamental morphology of the face [6]. This early craniofacial development is to a large degree linked to the role of sonic hedgehog (SHH) as a morphogen that patterns the anterior vertebrate embryo and in particular the facial structures that originate from the first branchial arch [7]–[9].

Comb development has been a subject for very few studies since the transplantation experiments by Irvine E Lawrence in 1971 until recently, when the molecular nature of the *Pea-comb* mutation was revealed [10], [11]. Pea-comb is caused by a copy-number mutation; an expansion of a duplicated sequence located near regulatory sequences in intron 1 of *SOX5*. The mutation causes transient and ectopic expression of SOX5 in mesenchyme where the comb will subsequently develop [10]. *SOX5* belongs to group D SRY-related HMG-box (SOX)-genes (*SOX5*, –*6* and –*13*), which often have overlapping expression patterns and are known to cell-autonomously control cell fate [12]. Mesenchymal cells condense and are determined towards chondrocytes under the control of SOX5, together with SOX6 and –9 [13]. Moreover, SOX5 promotes generation of cranial neural crest and controls neurogenesis [14].

In our work we have studied the mechanisms of chicken comb morphogenesis and by exploring the *Pea-comb* mutant, a role for SHH in comb patterning and morphogenesis was exposed. The result shows that the SHH receptor expression was decreased in Pea-comb mesenchyme and hence we hypothesized that the capacity of the mesenchyme to respond to SHH-morphogenic signals is changed directly or indirectly by the ectopic SOX5 expression. By experimentally blocking SHH signalling, the single-comb phenotype could be transformed into a Pea-comb-like phenotype. These results show that SHH signalling regulate the development of the single comb and suggest that the capacity of the mesenchyme to respond to SHH is distorted by the ectopic SOX5 expression. The results illustrate the importance of strict spatial and temporal regulation of the mesenchyme competence during patterning of the comb-ridge and for the subsequent comb morphogenesis.

## Materials and Methods

### Ethics Statement

This study was carried out in strict accordance with the recommendations in the Guide for the Care and Use of Laboratory Animals of the Association for research in vision and ophthalmology. The protocol was approved by Uppsala försöksdjursetisk nämnd (Permit number: C4–12).

### Animals

The age of embryos is denoted by their embryonic day (E) and were staged according to Hamburger and Hamilton 1951 [15]. Pea-combed chickens (*P/P*) were sampled in an INRA experimental line (CH1) derived from a French local layer breed and a dwarf Bantam and is the same as used in our previous study [10]. Note that the CH1 line does not carry the sex-linked dwarf, *DW* allele. Birds are maintained at INRA-Centre de Recherches de Tours in the PEAT experimental facility. The single-combed wild-type chickens were sampled in another INRA experimental line (Nunukan) originating from a cross between Indonesian chickens and a commercial brown-egg layer (INRA) and were also obtained from local breeds of White Leghorn (OVA production, Västerås, Sweden). The chicken lines are depicted in figure 1. For each genotype, 8 to 9 females were inseminated with mixed semen of 2 to 3 males and fertile eggs were collected daily. Subsets of eggs were incubated sequentially in order to obtain 6 embryos for each desired embryonic stage for gene expression studies, and 4 to 5 embryos for each embryonic stage for histology. White Leghorn embryos were used for cyclopamin treatments. Optimally the pea-comb mutation should have been studied in the same genetic background as the single-combed animals in this experiment.

### Quantitative Reverse-Transcription PCR (qRT-PCR)

RNA was extracted from comb tissue from E6 to E18, Pea*-* and single-comb embryos using TRIzol (Invitrogen, Grand Island, NY, US). RNA was treated with DNase (1 µg/µl) and cDNA was prepared from 1 µg RNA by reverse-transcription (MultiScribe RT, Applied Biosystem, Carlsbad, CA, US) and random hexamer priming. The qRT-PCR analysis was performed using IQ™ SyBr Green Supermix (Biorad, Hercules, CA, US) with primers designed by using Primer Express v2.0 (Applied Biosystem, Carlsbad, CA, US), checked for PCR efficiency, linear dynamic range and specificity. The mRNA levels were normalized to β-actin mRNA levels. The use of β-actin for normalization purposes was validated by testing for the most stable mRNA expression of TBP, β-actin, ß-2-microglobulin and glyceraldehyde 3-phosphate dehydrogenase over the developmental stages using geNorm [16]. Primer sequences and corresponding accession numbers of target sequences are listed in Table S1A. Expression levels were calculated from cycle threshold (Ct) and the 2^-ΔΔCt^ method [17]. The normalized amplification levels of Pea- and single-comb relative to the ß-actin amplification levels are shown, and differences were tested by using one-way analysis of variance (ANOVA) followed by Tukey’s range test as indicated in figure legend. Data were analysed through the use of Ingenuity Pathways Analysis (Ingenuity Systems, www.ingenuity.com).

### Alcian Blue Staining

Staged embryo heads were fixed in Bouin’s solution for one hour at room temperature and transferred to 30% sucrose in PBS overnight at 4°C. The heads were frozen in OCT freezing medium (Sakura Tissue-Tek, Alphen aan den Rijn, Netherlands) and sectioned. Ten µm sections were rinsed in PBS, post-fixed in Bouin’s solution for 10 min. at room temperature, washed five times in 70% ethanol, 0.1% NH_4_OH, washed twice in 5% acetic acid for 5 min. and stained with 0.05% Alcian blue 8GX (C.I. 74240, HistoLab Products AB, Göteborg, Sweden) in 5% acetic acid for 20 min. at room temperature. The sections were washed twice in 5% acetic acid 5 min., rinsed in 100% methanol and mounted in 90% glycerol.

### Immuno- and *in situ* Hybridisation Histochemistry

Heads were fixed in 4% paraformaldehyde in PBS for one hour at 4°C, transferred to 30% sucrose in PBS overnight at 4°C, frozen in OCT freezing medium and sectioned 10 µm with a cryostat. The sections were washed in PBS and used for both immuno- and *in situ* hybridisation histochemistry. For immunohistochemistry: sections were blocked (PBS with 1% fetal calf serum, 0.1% Triton-X, 0.02% Thimerosal) before addition of primary antibodies in blocking solution and incubated overnight at 4°C. The slides were washed 3 times for 5 min. in PBS before incubation secondary antibodies in blocking solution in room temperature for two hours. The slides were washed 3 times 5 min. with PBS before mounting. Antibodies: Sox5; Abcam Ab26041 Rabbit 1∶1000, SHH; Developmental Studies Hybridoma Bank 5E1 Mouse 1∶100 and ETS1; Abcam ab10936 Mouse 1∶200. Secondary antibodies (Alexa Fluor, Invitrogen, Grand Island, NY, US) were made in donkey. *In situ* hybridization analysis was performed as previously described [18], [19]. A cRNA PTCH1 probe (841 bp) [20] was made using the DIG RNA labelling kit (Roche, Basel, Switzerland) and hybridized to sections over night at 68°C under conditions containing 50% formamide and 5X SSC. The DIG labelled probe was detected by using alkaline-phosphatase and BCIP/NBT (Roche, Basel, Switzerland) for 2–5 hours at 37°C. Images from immuno and *in situ* hybridization analysis were captured by using a Zeiss Axioplan2 microscope and Axiovision software (4.8, Carl Zeiss GmbH, Hamburg Germany).

### Experimental Disruption of SHH Signalling

Cyclopamine (1 mg/ml, C4116, Sigma-Aldrich, St. Louis, MO, US) was prepared in a 45% solution of 2-hydroxylpropyl-β-cyclodextrin (HBC, H107, Sigma-Aldrich) in PBS. Fertilized White Leghorn eggs were windowed at E4, 5, 6 and 7, and 20 µl cyclopamine solution was injected beneath the vitelline membrane into the perifacial region with a glass micropipette. Control embryos received equal volume of the HBC/PBS solution. Eggs were sealed and incubated to the desired age E12–18. Embryos were examined for morphological changes to the comb region following fixation in 4% paraformaldehyde in PBS for one hour at 4°C. Fixed heads were incubated overnight in 30% sucrose in PBS at 4°C, embedded in OCT freezing medium, frozen and cryo-sectioned. Non-parametric Mann-Whitney U-test was used to test the occurrence of malformed comb morphology in cyclopamine and control treated animals.

## Results

### Facial Cartilage Development is Normal in Pea-combed Chicken

The beak or head of E12, E18, 1, 4/6 and 35 weeks old Pea-comb (Fig. 1A, C, K, L, N-P and R) and single-comb (Fig. 1B, D, J, M, Q and S) chickens is shown to illustrate the morphological development of the Pea-comb. We analysed cranial and beak cartilaginous structures in embryonic Pea- and single-comb heads by Alcian blue staining. Comparison of external morphology, Alcian blue stained whole mounts and sections (Fig. 1E–H) did not display any notable differences between the comb-types in the external or internal beak or facial structures other than the comb and wattles.

### Analysis of Down-stream Candidate Genes in Pea- and Single-comb Tissue

Previous analysis of comb development indicated that the mesenchyme might direct comb development [4], [5]. The ectopic expression of SOX5 in the Pea-comb mesenchyme [10] at the time of comb formation further supported this hypothesis (Fig. 1I). To identify gene expression associated with the Pea-comb phenotype and ectopic SOX5 *expression*, we analysed samples containing comb epidermis and underlying comb mesenchyme from E7, E12 and E18 heads of Pea- and single-combed animals. Embryonic day 7 is before the first visible comb-ridge but after ectopic SOX5 expression in Pea-comb mesenchyme (Fig. 1I), E12 is the time when the divergent morphology of comb types is visible (Fig. 1A, B) and at E18 the phenotype is established (Fig. 1C, D). SOX5 expression is higher in Pea-comb at E7, E9 and E12 (Fig. 1I). We selected genes that were related to known SOX5 functions, such as genes playing roles in chondrocyte and neural crest development as well as in extra cellular matrix synthesis (listed in Table S2).

**Figure 2:**
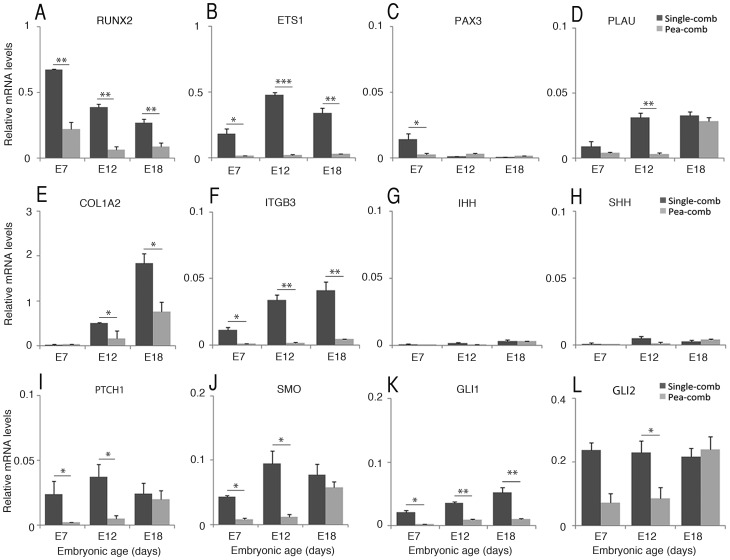
Candidate gene expression analysis in Pea- and single-comb tissue. Bar graphs with qRT-PCR analysis data for (A) RUNX2, (B) ETS1, (C) PAX3, (D) COL1A2, (E) ITGB3, (F) IHH, (G) SHH, (H) PTCH1, (I) SMO, (J) GLI1 and (K) GLI3. Bar graph data are normalized to ß-actin mRNA levels and is relative to the ß-actin mRNA level in Pea- and single-comb tissue respectively. Bar graphs are mean±s.e.m., ANOVA, n = 6 (single-comb) n = 5 (Pea-comb). * p<0.05, **p<0.001.

Among 35 selected genes; *RUNX2*, *ETS1*, *PAX3*, *COL1A2*, *PLAU* and *ITGB3* were differentially expressed. The mRNA levels for these genes were lower in Pea-comb than in single-comb (Fig. 2A–F). The analysed genes, their expression levels in single-comb tissue and fold difference compared to Pea-comb are listed in Table S2. Notably, expression differences were not seen for the SOX5 partners; SOX6, 9 or 10, and immunohistochemistry did not reveal any co-expression of SOX6, 9 or 10 with the ectopic SOX5 expression (data not shown). Moreover, there were no expression differences seen for MEOX2, hyaluronic acid synthases (HAS2, 3), matrix-metallopeptidases (MMP1, 2, 13), cartilage collagen COL2A1, proteoglycan versican (VCAN) or for genes involved in neural crest development and derivatives (Snail (SNAI1), Slug (SNAI2), RHOA, TWIST, cKIT, MITF), BMP4 or Frizzled1 (FZD1).

**Figure 3:**
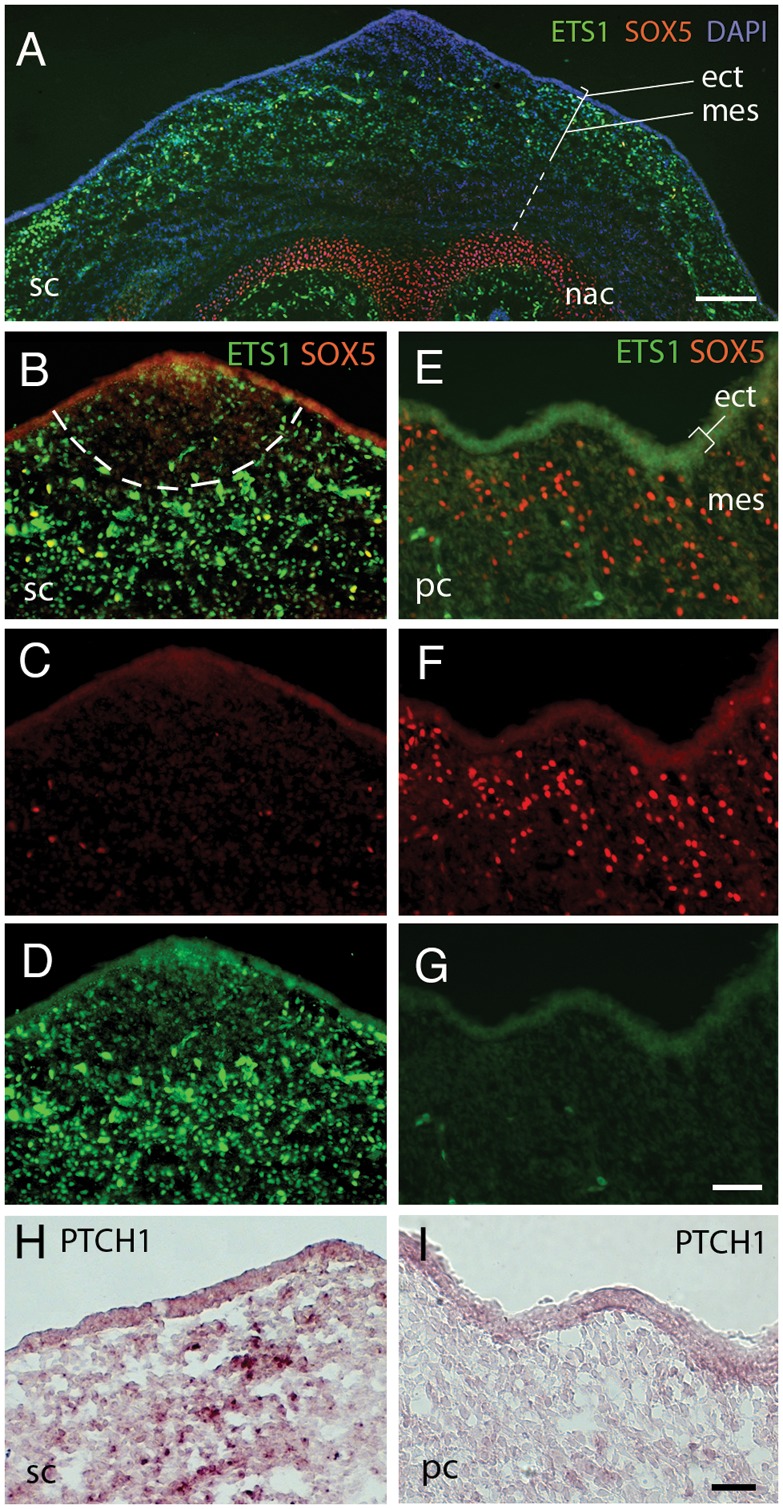
ETS1, SOX5 and PTCH1 expression in E9 Pea- and single-comb. Micrographs depicting immunohistochemical analysis of SOX5 and ETS1 expression and *in situ* hybridization analysis for PTCH1 mRNA in the E9 comb-region. (A) Low magnification fluorescence micrograph showing the single-comb region with SOX5 in nasal cartilage and ETS1 in the dermal mesenchyme. DAPI staining visualises the mesenchymal condensation under the comb-ridge. Sub-dermal mesenchyme is indicated by the dashed straight line. (B) Single-comb-ridge with staining for SOX5 (red) and ETS1 (green). The mesenchyme condensation is delineated by the dashed line and correlates with lower ETS1 expression. (C, D) Separate red and green fluorescence signals for image shown in B. (E) Pea-comb-ridge with staining for SOX5 (red) and ETS1 (green). (F, G) Separation of fluorescence signals shown in E. (H, I) PTCH1 in situ hybridisation analysis of (H) single- and (I) Pea-comb-ridge. ect; ectoderm, mes; dermal mesenchyme, nac; nasal cartilage, pc; Pea-comb, sc; single-comb. Scale bars in A, I are 100 µm and in G 50 µm also valid for B–F.

RUNX2 is essential for the commitment of multipotent mesenchymal cells to osteo- and chondroblastic lineages and for the formation of prechondrogenic mesenchymal condensations [21], [22]. RUNX2 works together with ETS1 as transcriptional activator [22], [23] upstream of COL1A2, PLAU and ITGB3 [24], and the reduced levels in Pea-comb are consistent with decreased RUNX2 expression. Immunohistochemistry for ETS1 and SOX5 revealed robust ETS1 labelling in dermal mesenchyme in E9 single-comb. The cells just underneath the central comb-ridge corresponding to mesenchymal cell condensation, visualized by the DAPI staining, showed weaker ETS1 labelling than the surrounding cells (Fig. 3A). Strongly SOX5-labelled cells were seen in nasal cartilage as well as in a few scattered cells in the mesenchyme and in the ectoderm of both single- and Pea-combed chicken as described previously [10] (Fig. 3A,C) while the Pea-comb tissue exhibited strong ectopic SOX5 expression in the dermal mesenchyme [10] (Fig. 1I, 3E). The reduction of ETS1 expression shown by qRT-PCR in Pea-comb (Fig. 2B) was confirmed by the immunohistochemistry (Fig. 3E, G).

### Sonic and Indian Hedgehog Receptors are Down-regulated in Pea-comb

An Ingenuity Pathway Analysis of the expression profiles of single- and Pea-comb suggested that SHH/Indian hedgehog (IHH) signalling could be involved (Fig. S1). IHH is expressed locally in mesenchymal condensations and regulates the expression of RUNX2 [25]. We analysed the expression of IHH, SHH, their receptors Patched (PTCH1), Smothened (SMO) and down-stream effectors GLI1 and -2 (Fig. 2G–L). The levels of IHH and SHH mRNA were just above background and did not differ significantly between the comb types at the analysed time points (Fig. 2G, H). In contrast, PTCH1, SMO, GLI1 and -2 mRNA levels were lower in Pea-comb tissues than in wild-type tissue (Fig. 2I–L). *In situ* hybridisation confirmed the reduced PTCH1 expression in E9 Pea-comb (Fig. 3H, I). These results imply that the Pea-comb ridge mesenchyme has altered SHH/IHH signalling and that the mesenchyme may have a reduced capacity to respond to SHH/IHH. The SHH receptor expression has previously been shown to be regulated by SHH itself [26] and we therefore analysed the SHH expression in the facial region of E4 single- and Pea-comb embryos.

### SHH Expression in Facial Ectoderm

**Figure 4:**
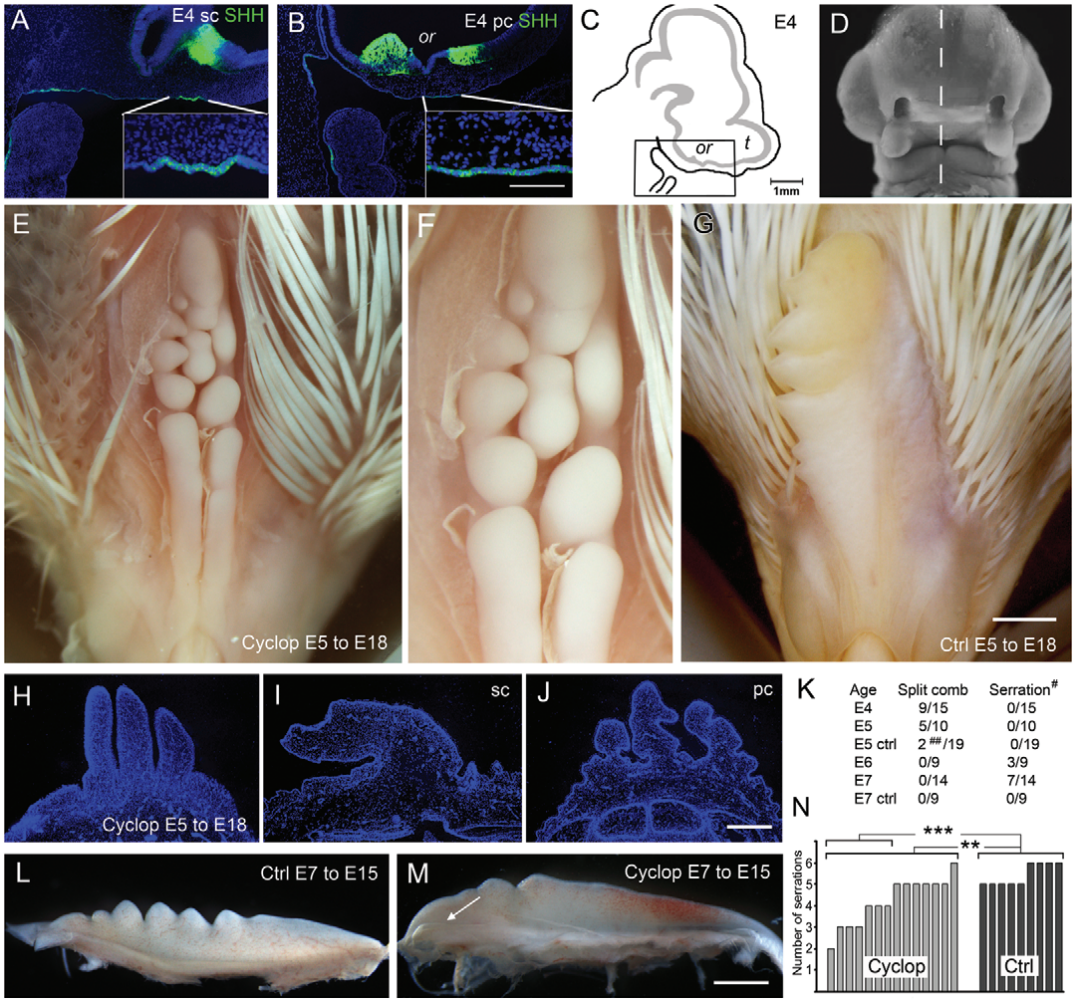
SHH expression and effects of cyclopamine treatment during comb formation. SHH expression was analysed by using immunohistochemistry of Pea- and single-comb E4 embryos. Fluorescence micrographs of (A) sagittal section of a single-combed and (B) a Pea-combed E4 head labelled for SHH. (C) Schematic illustration of the region shown in sections depicted in A–B. (D) Frontal view of the facial region of an E4 single-combed chicken head with the plane of sections in A–C indicated by a dashed line. (E) Dorsal view of the forehead of an E18 single-combed chicken treated with cyclopamine at E5. The beak is pointing down in the image. Note the comb that is split in three rows of serration in the caudal part. Some feather anlagen were removed to better display the comb. (F) Magnification of the affected comb-region depicted in E. (G) E18 single-comb control chicken. (H–J) Fluorescence micrograph of DAPI stained cross section of (H) the cyclopamin-treated comb depicted in E, (I) an E18 single-comb and (J) a E18 Pea-comb. (K) Table with the number of animals affected when treated at E4– E7 by cyclopamine or control with split comb or affected serrations. ^#^ Number of individually distinguishable points or serrations. ^##^ Two control embryos were affected by other head and intestine malformations. Side-view of serrations of E15 single-combs treated at E7 with (L) HBC/PBS control and (M) cyclopamine. Arrow indicates the posterior part of the comb with a lateral expansion. (N) Bar-graph showing the effect on serration seen at E15 in cyclopamine or HBC/PBS control-treated single-combs at E7. Non-parametric Mann-Whitney U-test, n as indicated in the figure * *p*<0.05 ** *p*<0.01, *** *p*<0.001. Scale bar in C is 100 µm also valid for A and B, bar in G is 1000 µm also valid for E, bar in J is 400 µm also valid for H and I, bar in M is 250 µm also valid for L. E; embryonic day, Ctrl; Control, Cyclop; Cyclopamine, or; optic recess, pc; Pea-comb; sc; single-comb, SHH; Sonic hedgehog, t; telencephalon.

The facial region has been extensively studied and SHH is expressed in distinct craniofacial organizing centres: (i) strong expression in the ventral midline neuroepithelium of the prosencephalon, (ii) in ectoderm of the frontonasal process that will give rise to the upper beak and comb-ridge and (iii) in ectoderm of the maxillary process in the first branchial arch [27], [28]. Both the spatial and temporal aspects of the expression are important for patterning of the developing facial prominencies [29]. Immunohistochemistry was used to study SHH expression domains in E4 (st23) embryos with special focus on the frontonasal ectoderm. Similar expression patterns were seen in single-comb and Pea-comb embryos (Fig. 4A–D).

### Perturbation of SHH Signalling Elicits a Pea-comb-like Phenotype

We hypothesised that ectopic expression of SOX5 in the *Pea-comb* mutant could intervene with SHH signalling in the comb-mesenchyme. A negative regulation of PTCH1 has been suggested by the observed increase of PTCH1 expression in SOX5 knock-out mice [30]. Furthermore, the lower levels of PTCH1, SMO, GLI1 and GLI2 expression in Pea-comb compared to single-comb suggested that attenuated IHH/SHH signalling contribute to the development of the Pea-comb phenotype and that SHH may contribute to the patterning of the wild-type single-comb.

Cyclopamine disrupts SHH signalling by binding to SMO [31] and has dose- and stage-specific teratogenic effects on face development [28]. Reports on effects of either SHH or cyclopamine on comb development could not be found. We tested if SHH signalling regulated single-comb morphogenesis by treatment with cyclopamine at the stages that followed the formation of the facial primordia and thus preceded that of the comb-ridge. Fertilized eggs were windowed and single-comb embryos were treated at E4, E5, E6 and E7 and the comb morphology was studied at E15 and E18. We saw clear effects on the comb morphology after cyclopamine treatment compared to controls, with split comb-ridge and reduced number of comb serrations (Fig. 4E–N). The survival after injections was similar in the cyclopamine and control animals (approx. 50%) with 76 surviving embryos at E15 (Fig. 4K). The effects of cyclopamine followed a temporal pattern; early treatments at E4–5 (st24–26) produced full or partial split of the anterior comb-ridge with variable effects on the serrations; late treatments at E6–7 (st27–29) also had effects on the comb serrations but without any split of the anterior comb-ridge (Fig. 4L, M). The posterior part of the ridge was often laterally expanded (Fig. 4M). Some combs had a mixed effect with half the comb being split and with disturbed serrations or three rows of serrations seen at the posterior part of the comb (Fig. 4E, F). Size and number of the serration points were affected. The effects of cyclopamine treatment differed significantly from the effects seen after control treatments (Fig. 4N). These results show that the lateral and longitudinal morphology of the single-comb is established under the regulation of a cyclopamine-sensitive signal from E4 to E7. The number and appearance of the comb serration are under the regulation of similar signals and the patterning occurs in an anterior to posterior direction.

## Discussion

The identification of the *Pea-comb* mutation has given a tool to investigate how the chicken comb is formed and how the morphology develops. Pea-comb is a dominant trait, although with variable expression, and as such the small irregular comb shape can differ not only between homo- and heterozygous birds but also among different Pea-combed chicken strains [10]. The *Pea-comb* mutation is a copy number expansion in the vicinity of evolutionary conserved sequences in *SOX5* intron 1 that is causing transient ectopic SOX5 expression in mesenchyme underlying the developing comb and wattles. This means that a “foreign” transcriptional regulator with potent and pleiotropic effects is expressed out of its normal context, in the comb mesenchyme. By analysis of candidate gene expression we identified among other genes a reduced expression of the SHH receptors; PTCH1 and SMO in the comb mesenchyme of the Pea-comb chicken, implying attenuated SHH signalling. By experimentally disrupting SHH signalling using cyclopamine, we showed that a single-combed chicken can be transformed into a Pea-comb-like phenotype. These morphological changes are consistent with previous studies on the facial development in which disrupted or excess SHH truncates, elongates or even produces extra facial prominencies [29]. Hence, SHH plays a role in the mediolateral axis formation of the mid and upper face including the region where the comb develops.

SHH is expressed from early stages in the ectoderm of the developing frontonasal processes and of the first branchial arch [32]. PTCH1 is upregulated by SHH in both ectoderm and underlying mesenchyme [20]. Our results suggest that the ectopic SOX5 expression in Pea-comb interferes with the PTCH1 and SMO-expression in comb mesenchyme. Such block is consistent with the negative regulation of PTCH1 expression downstream of SOXD (*SOX5*) genes [33]. The reduced PTCH1 and RUNX2 expression is also consistent with studies showing that IHH regulates osteoblast differentiation of mesenchymal cells via PTCH1 and SMO, where increased IHH up-regulates the expression and function of RUNX2 [24], [25]. The reduced RUNX2 may therefore be a result of the ectopic SOX5 expression intervening with PTCH1, SMO and GLI signalling in the Pea-comb mesenchyme. We attempted to express SOX5 ectopically in the comb mesenchyme by using retrovirus vector or *in ovo* electroporation of SOX5 expression vector, but we were not able to achieve expression in the mesenchyme. The mesenchyme is derived from cranial neural crest that migrates into the region prior to comb formation [34]. SOX5 plays a role in the generation and migration of cranial neural crest and ectopic expression in the neural crest is known to affect its development [14]. This may explain the complications with this seemingly trivial experiment. Weather the reduced PTCH1 expression is a direct effect of the ectopic SOX5 expression in the *Pea-comb* mutant or not remains to be elucidated. One may argue that the perfect experiment is present in the *Pea-comb* mutant where the specific mutation causes the ectopic SOX5 expression. An approach to conditionally knock-down PTCH1 or SMO expression in comb mesenchyme would give evidence for what the pharmacological cyclopamine treatment has revealed.

As commented previously, RUNX2 functions during formation of prechondrogenic mesenchymal condensations [35] and is one of the major orchestrating transcription regulators in the commitment of cells to osteo- and chondroblastic lineages [21]–[23], [36]. Deficiency in RUNX2 is associated to cleidocranial dysplasia in humans with the characteristic facial features including a prominent forehead, wide-set eyes (hypertelorism), a flat nose and a small upper jaw [35], [37], showing its importance in the medio-lateral face development.

The cyclopamine-induced comb phenotype was similar but not identical to that of the Pea-comb (Fig. 1, 4E–N). The ectopic SOX5 expression is likely to have effects on additional genes and temporal differences in the proposed attenuation of the SHH signalling in Pea-comb and cyclopamine treatment may contribute to the phenotypes. The injection time of cyclopamine was important. Treatment at E4–E5 split the anterior comb-ridge while treatment at E6 affected the posterior part with serration defects and lateral expansion of the comb. (Fig. 4K, L–N). The Pea-comb does not exhibit any split of the anterior ridge. Instead, the posterior part is affected showing a reduced comb size and number of serration points as well as the characteristic 3-split. The Pea-comb phenotype is more similar to the late experimental phenotype.

Several other factors regulate facial morphogenesis. The growth and size of the major facial prominences are under the influence of WNTs, bone morphogenetic factors and fibroblast growth factors [38], [39]. The general size-reduction of the Pea-comb is likely to reflect a reduced overall growth of the comb-tissue and similar mechanisms as seen in the facial prominences are likely to contribute to the secondary growth of the comb. The sex-specific contributions to comb size is also seen in the Pea-combed chickens (Fig. 1P–S).

The reduction of ETS1 expression in Pea-comb was visualised by immunohistochemistry (Fig. 2B, 3E). ETS1 interacts directly with the RUNX2 expression regulatory network [23]. ETS1 is expressed in neural crest cells in the first and second pharyngeal arches [40], [41] and influences cranial neural crest cell migration and augments epithelial-mesenchymal transition induced by Slug [SNAI2] [42]. It coordinates changes in cell adhesion and degradation of extra cellular matrix and is classified as having tissue remodelling activities both during normal embryogenesis and tumour metastasis [43]. Effects elicited by reduced expression of ETS1 but also of the known down-stream genes of ETS1, integrin B3 (ITGB3) and the urokinase-type plasminogen activator (PLAU), are consistent with the changes seen in the Pea-comb. The condensed mesenchyme with low ETS1 expression just underneath the E9 comb-ridge in single-comb seems to have expanded laterally in Pea-comb to include a larger region of the mesenchyme (Fig. 3A–G).

The importance of ectoderm-mesenchymal interactions have long been acknowledged, much based on tissue recombination experiments of embryonic ectoderm and mesenchyme in different sources [44]. In beginning of the 60′s, Irving E Lawrence addressed the mechanisms behind the different comb traits as reported by William Bateson [1] [45]. Lawrence’s results proposed that the comb mesenchyme determines the structural organization of the comb while the ectoderm assumes more of a passive role. He also concluded that his transplantation data indicated that no identifiable comb tissues differentiated from grafts made earlier than stage 24 (E4) [4], [5]. These results are now closer to an explanation in the light of the present data. We propose that the ectopic SOX5 expression modifies the competence of the mesenchyme, which leads to the conspicuous re-organisation of the comb structure seen in Pea-comb (Fig. 1). This hypothesis is consistent with recent results showing that the neural crest mesenchyme actively participates in patterning of the ectoderm in the facial region of the chick embryo [46].

In another seminal publication, William Bateson reported together with Reginald C Punnet in 1905, the first example of epistatic genetic interaction [47]. They demonstrated that the Walnut-comb phenotype is caused by the combined effect of the *Pea-comb* and *Rose-comb* genotypes. *Rose-comb* is also dominantly inherited and causes distorted comb morphology. The Rose-comb is large and “pointy” with lateral expansion of the comb but without the three initial comb-ridges. We have recently identified the *Rose-comb* mutation as a 7.4 M base pair inversion on chromosome 7 [48]. Interestingly, the *Rose-comb* mutation, like the *Pea-comb*, generates ectopic expression in comb mesenchyme. The inversion positions the Mnx-class homeodomain protein gene *MNR2* [49] in an alien genetic position that drives ectopic MNR2 expression in the comb mesenchyme [48]. Mnx-transcription factors act as transcriptional repressors and specify cell fate and differentiation [50]. The Walnut-comb is larger than the Pea-comb and lacks the numerous points seen in the Rose-comb. The epistatic interaction of the *Pea-* and *Rose-comb* mutations can be explained by the co-expression of SOX5 and MNR2 in mesenchymal cells although with different but overlapping temporal windows. The combined ectopic expression of two fundamentally fate-determining transcription factors will alter the mesenchyme; both its competence to respond to patterning signals and how the mesenchyme will develop.

The present study does not only add to our knowledge how the comb and facial structures are formed, it also adds how variation in facial morphology may be achieved. The results indicate that epithelial-mesenchymal interactions are important for comb development, and it is plausible that similar interactions occur and contribute to the mediolateral patterning of other facial structures. Mutations, such as the *Pea-comb* mutation, or genetic polymorphisms that modify the competence even just slightly or shift the temporal profile minutely may contribute to the inherited morphological variability of the face.

## Supporting Information

Figure S1 Ingenuity pathway analysis of candidate gene expression in the E7 comb region.
(PDF)

Table S1 qRT-PCR primer sequences.
(PDF)

Table S2 Comparison of Pea- and single-comb gene expression; candidate genes.
(PDF)
