# Supplementary material for: Sonic Hedgehog-Signalling Patterns the Developing Chicken Comb as Revealed by Exploration of the Pea-comb Mutation
Source: PLoS One. 2012 Dec 5;7(12):e50890. doi: 10.1371/journal.pone.0050890 (PMC3515514; doi:10.1371/journal.pone.0050890)

<u>Supplemental figure S1.</u> Henrik Boije et al. <u>Ingenuity pathway analysis of candidate genes.</u> The network is a graphical representation of the molecular relationships between the analysed candidate molecules in the E7 comb. Molecules are represented as nodes, and the biological relationship between two nodes is represented as an edge (line). All edges are supported by at least 1 reference from the literature, from a textbook, or from canonical information stored in the Ingenuity Pathways Knowledge Base. Human, mouse, and rat orthologs of a gene are stored as separate objects in the Ingenuity Pathways Knowledge Base, but are represented as a single node in the network. The intensity of the node color indicates the degree of up- (red) or down- (green) regulation. Nodes are displayed using various shapes that represent the functional class of the gene product. The network was generated through the use of Ingenuity Pathways Analysis (Ingenuity Systems, www.ingenuity.com).

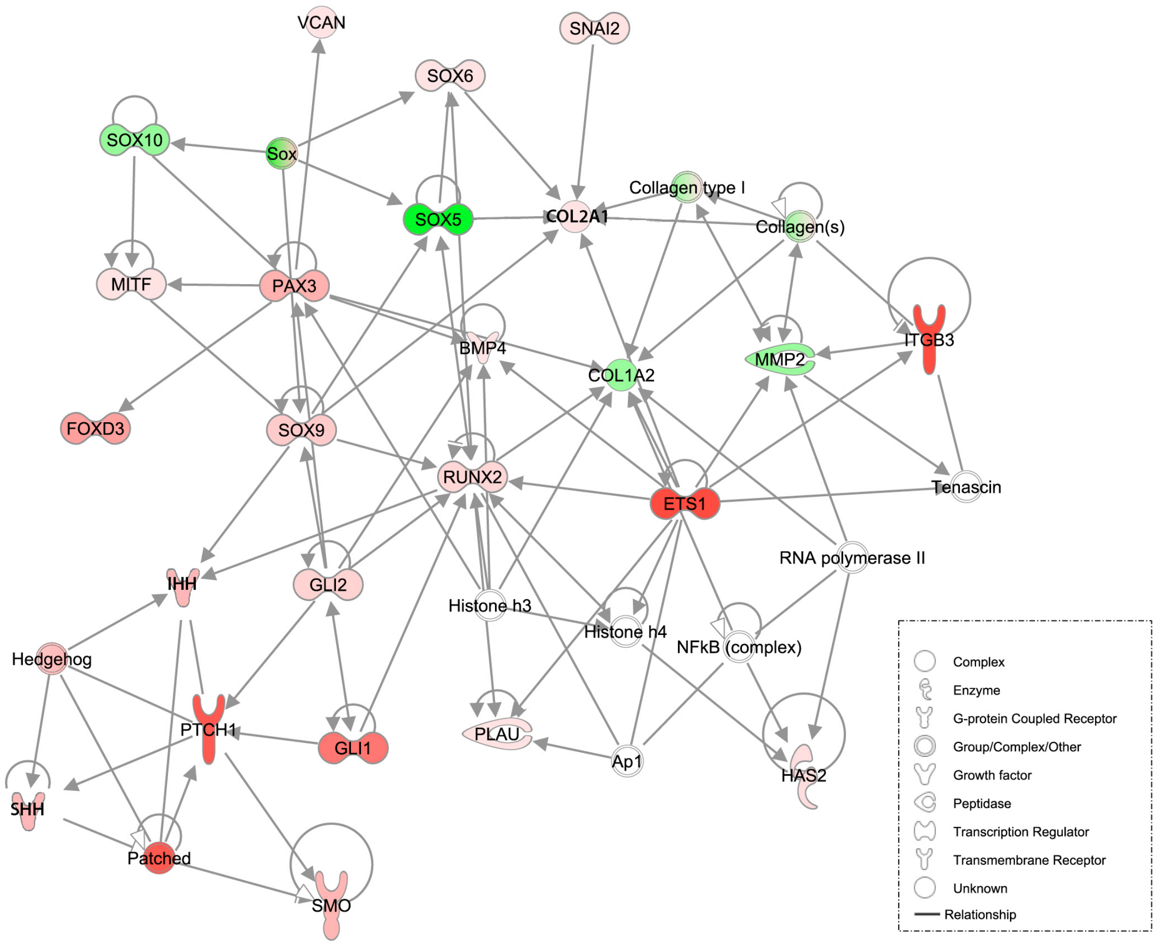

Supplement: Figure S1 — Ingenuity pathway analysis of candidate gene expression in the E7 comb region. (PDF) [file pone.0050890.s001.pdf]
