# Supplementary material for: Sonic Hedgehog-Signalling Patterns the Developing Chicken Comb as Revealed by Exploration of the Pea-comb Mutation
Source: PLoS One. 2012 Dec 5;7(12):e50890. doi: 10.1371/journal.pone.0050890 (PMC3515514; doi:10.1371/journal.pone.0050890)
Supplement: Table S1 — qRT-PCR primer sequences. (PDF) [file pone.0050890.s002.pdf]

Supplemental Table S1 (Boije et al, Sonic hedgehog signalling patterns the developing chicken comb as revealed by exploration of the Pea-comb mutant)

Supplemental Table S1: List of qRT-PCR Primers

**Genes**	**Primer sequence (5́-3́)**<br>**F- forward, R-reverse**	**Ensemble ID**
ARHGEF12	F-CGACTTCGATCCGACAGATAGC<br>R-CCGTTGGATAACAACACACCG	ENSGALG00000006663
BMP4	F-TTTACGAAGTGATGAAGCCGCT<br>R-CTCACATCAAAGGTCTCCCAGC	ENSGALG00000012429
cKIT	F-TCATCGGTGCCATCCTTTGA<br>R-CTTGTCACCATTGCTGGATGC	ENSGALG00000013925
COL1A2	F-GCTTTGTGGATACGCGGATTT<br>R-TGTGGCCCTTTGTCTCCTCTAG	ENSGALG00000009641
COL2A1	F-TTCACCTACAGCGTCTTGGAGG<br>R-GATATCTACAATGGACAGGCGCG	ENSGALG00000013587
DCT	F-TGAGGAAGCCACCAGTTGTCA<br>R-GGTGGATGGTAGTCTTTGCAC	ENSGALG00000016899
EFEMP1	F-TGCCCCGTATCAAACACCAT<br>R-TGGTTGCCTGAATCTGGAAGAT	ENSGALG00000007908
ETS1	F-GCAGAAAGAAGAGGCAAAACCA<br>R-GCGTGCTCGATGCCATAACTA	ENSGALG00000001143
FEZF2	F-GTGCACCATTTGCAACAAAGC<br>R-TCCTTTGCCGCAAGTGACA	ENSGALG00000007184
FOXD3	F-ACAACCTCTCGCTCAACGACTG<br>R-CGTTGTCGAACATGTCCTCAGA	ENSGALG00000012644
FZD1	F-CCATGAGCCCTGACTTCACTGT<br>R-AGTTGAGCGTTTTCCCCGA	ENSGALG00000009064
GLI1	F-CCTCTGACAGCCAAATACCCAG<br>R-TTTCCCATCCTCCTTCTCCAG	ENSGALG00000014639
GLI2	F-CTGCAGACGATGATCAGGACCT<br>R-GGCAGATAAATGGCCGTAGGA	ENSGALG00000011630
HAS2	F-TGCGTACAGTGCATCAGTGGA<br>R-AGCCCAGACTTAGCACTCGGTT	ENSGALG00000016394
HAS3	F-CAGTTCATCCACACGGAGAAGC<br>R-GGTGCTCCAGGAAAGCAAAGA	ENSGALG00000000630
IHH	F-GCAACAAGTACGGCATGCTTG<br>R-TACCTGACTTGACGGAGCAGTG	ENSGALG00000011347
ITGB3	F-CAACCTTCGCATCGGCTTT<br>R-GGCAAGCACTTTTCCCCAAT	ENSGALG00000000379
MEOX2	F-GAGGAAAAGCGACAGCTCAGAT<br>R-TCTCTGATTTGCTCCTTGGTGA	ENSGALG00000010794
MITF	F-ACATGGACTGTCCCTTGTTCCA<br>R-GGTCTTGGTTGCAGTTGTCCAG	ENSGALG00000007679

MMP1	F-AGTTCCAGTTTGATCCTCGTGC<br>R-TGCAGTGTGCATGTGGAAAAG	ENSGALG00000019061
MMP13	F-TGCTGGTCTTCAAGGACAGGTT<br>R-TTTGTTTGGAAGCTCCGGC	ENSGALG00000017183
MMP2	F-TAGATGATGCCTTTGCCCGAG<br>R-TGTTCCCATCGGCCAAAATT	ENSGALG00000003580
PAX3	F-CCCAGTCTGATGAAGGCTCTGA<br>R-TTTCCAGCTCTTCCAGTTGCTC	ENSGALG00000005246
PAX7	F- CACATCCGCCATAAGATCGTG<br>R-GGTACCTGCAGAGGATTTTGGA	ENSGALG00000003782
PLAU	F-AGGAATGGCAGAAGATCCAGG<br>R-CCAGTCCAAGTTGCAGAGCAT	ENSGALG00000005086
PTCH1	F-TGCAGAGCCACGATCAGAAAT<br>R-AGCAAATCCCTTGTTGAGCTTG	ENSGALG00000012620
RHOA	F-AGCGTGGCTTCAGTGCTATCA<br>R-GCTGAATGGAACGTGGTCATTT	ENSGALG00000003806
RUNX2	F-GCTAAACCCAAACTTGCCCAA<br>R-GGACTCATCCATCCTGCCACTA	ENSGALG00000016706
S100B	F-TGAAGAAATCGGAGCTGAAGGA<br>R-TCCAGTGCCTCCATGACTTTG	ENSGALG00000006217
SHH	F-CCCCAAATTACAACCCTGACAT<br>R-TTCATCACCGAGATCGCCA	ENSGALG00000006379
SMO	F-CATCGCTACGCAGACCAATAAA<br>R-ATGCCAGTGCCAAACATGG	ENSGALG00000007910
SNAI1	F-CTGCACCCATCTGGACATCTTT<br>R-GCGAATGCCAACACTGTGATC	ENSGALG00000008018
SNAI2	F-TCTCCAGACCCTGGCTACTTCA<br>R-CCCTCAGATTGGATCTGTCTGC	ENSGALG00000015241
SOX5	F-AGGAACAGATTGCAAGACAACA<br>R-CTGCTGGATCTGTTGCTGAAG	ENSGALG00000013204
SOX6	F-ACTGTGGCTGAAGCTCGAGTCT<br>R-TCCTTAGCCCAAACCATGAAAG	ENSGALG00000006074
SOX9	F-TCCCCACATCGATTTCCGA<br>R-GCAGGTATTGGTCGAACTCGTT	ENSGALG00000004386
SOX10	F-CCCCCTGTCCCATCTGTTTAA<br>R-AAGGCTAAGGCTGACAGTGCAG	ENSGALG00000012290
SPAG6	F-AGATGATCCTGAACCCCGATG<br>R-GCTTCAACCACCAACTCTGCA	ENSGALG00000007892
TWIST	F-TGAGCCACTGAGGAGAGGAAGT<br>R-GCCAGTTATCCCCCCTAAAAAA	ENSGALG00000010862
VCAN	F-CAATATGAGAACTGGCGACCAA<br>R-TCTTGCAGGTGTAGGTCAGGTG	ENSGALG00000015624
