# Supplementary material for: Sonic Hedgehog-Signalling Patterns the Developing Chicken Comb as Revealed by Exploration of the Pea-comb Mutation
Source: PLoS One. 2012 Dec 5;7(12):e50890. doi: 10.1371/journal.pone.0050890 (PMC3515514; doi:10.1371/journal.pone.0050890)
Supplement: Table S2 — Comparison of Pea- and single-comb gene expression; candidate genes. (PDF) [file pone.0050890.s003.pdf]

Supplementary Table S2: (Boije et al) List of analysed genes, relative expression level[a)], fold difference, brief summary of function and references.

Gene	Embryonic age									Function	References
	E7			E12				E18			
	Level sc[a)]	FD	*p*	Level sc[a)]	FD	*p*	Level sc[a)]	FD	*p*		
ARHGEF12 (LARG)	bd			bd			bd			Vascular smooth muscle activation	(Ying, *et al*, 2006)
BMP4	0.029	1.55	0.32	0.095	1.87	0.20	0.227	1.02	0.22	Somitogenesis and patterning	(Monsoro-Burq, *et al*, 1996; Pourquie, *et al*, 1996)
COL1A2	0.026	0.86	0.45	0.506	**3.00**	**0.04**	1.839	**2.42**	**0.01**	Collagen type -I	(Merlino, *et al*, 1983; Nakata, *et al*, 1992; Retief, *et al*, 1985)
COL2A1	0.055	1.76	0.37	0.023	0.83	0.18	0.051	1.97	0.09	Collagen type -2	(Cheah, *et al*, 1985; Merlino, McKeon, de Crombrugghe, *et al*, 1983)
DCT	bd			bd			bd			Melanocyte development	(Pla, *et al*, 2004)
ETS1	0.182	**12.9**	**0.03**	0.478	**24.9**	**<0.01**	0.338	**11.5**	**<0.01**	Migration and differentiation of neural crest cells	(Fafeur, *et al*, 1997; Gao, *et al*, 2010; Kola, *et al*, 1993)
EFEMP1 (FBLN3)	0.056	4.29	0.29	0.287	3.87	0.13	0.151	0.93	0.43	Negative regulator of chondrocyte differentiation	(Wakabayashi, *et al*, 2010)
FEZF2	bd			bd			bd			Neurogenesis	(Kwan, *et al*, 2008)
FOXD3	0.0005	7.14	0.10	0.0004	0.24	0.16	0.0036	0.48	0.08	Melanocyte fate determination	(Thomas and Erickson, 2008; Tucker, 2004)
FZD1	0.039	2.15	0.27	0.069	3.61	0.15	0.108	0.84	0.30	Wnt receptor	(Hartmann and Tabin, 2000; Yu, *et al*, 2010)
GLI1	0.021	**10.1**	**0.04**	0.035	**4.01**	**<0.01**	0.052	**5.18**	**<0.01**	Hedgehog downstream signal	(Dai, *et al*, 1999; Marigo, *et al*, 1996)
GLI2	0.238	3.30	0.06	0.230	**2.69**	0.04	0.217	0.90	0.36	Hedgehog downstream signal	(Dai, Akimaru, Tanaka, *et al*, 1999; Marigo, Johnson, Vortkamp, *et al*, 1996)
HAS2	0.0036	2.48	0.38	0.0053	0.95	0.26	0.0037	1.52	0.13	Hyaluronic acid synthesis	(Itano, *et al*, 1999; Spicer, *et al*, 1997a; Spicer, *et al*, 1997b)
HAS3	bd			bd			bd			Hyaluronic acid synthesis	(Itano, Sawai, Yoshida, *et al*, 1999; Spicer, Olson and McDonald, 1997a)
IHH	0.0007	5.39	0.15	0.0017	3.52	0.08	0.0032	1.07	0.63	Indian hedgehog, morphogen	(Vortkamp, *et al*, 1996)
ITGB3	0.011	**12.9**	**0.03**	0.033	**24.4**	**<0.01**	0.041	**9.38**	**<0.01**	Integrin β3	(Lei, *et al*, 2011; Oda, *et al*, 1999; Rothhammer, *et al*, 2004)
KIT	0.002	2.03	0.11	0.001	0.43	0.30	0.004	1.81	0.06	Neural crest development	(Boissy and Nordlund, 1997)
MEOX2	bd			bd			bd			Somite formation Mesenchyme patterning and differentiation.	(Mankoo, *et al*, 2003; Reijntjes, *et al*, 2007)
MITF	0.007	1.62	0.30	0.005	0.43	0.07	0.027	1.53	0.14	Melanocytes development	(Opdecamp, *et al*, 1997)
MMP1	bd			bd			bd			Breakdown of ECM	(Klatt, *et al*, 2009)
MMP2	0.0231	0.86	0.42	0.0201	0.70	0.21	0.084	3.53	0.06	Breakdown of ECM	(Fichter, Korner, Schomburg, *et al*, 2006; Klatt, Paul-Klausch, Klinger, *et al*, 2009)
MMP13	bd			bd			bd			Breakdown of ECM	(Fichter, *et al*, 2006; Klatt, Paul-Klausch, Klinger, *et al*, 2009)
PAX3	0.014	**5.89**	**0.03**	0.001	0.35	0.07	0.0006	0.43	0.06	Neural crest migration	(Conway, *et al*, 1997)

PAX7	bd			bd			bd			Neural crest and somite development	(Rescan and Ralliere, 2010)
PLAU	0.008	2.10	0.32	0.031	**9.5**	**<0.01**	0.032	1.15	0.17	Urokinase-type plasminogen activator	(Iwasaka, *et al*, 1996; Rothhammer, Hahne, Florin, *et al*, 2004)
PTCH1	0.023	**12.0**	**0.02**	0.037	**7.66**	**0.03**	0.024	1.21	0.36	Hedgehog receptor	(Alcedo and Noll, 1997; Chen and Struhl, 1996)
RHOA	0.088	0.87	0.25	0.113	0.98	0.35	0.230	1.05	0.41	Regulation of chondrogenesis	(Woods and Beier, 2006)
RUNX2	0.671	**2.98**	**<0.01**	0.386	**6.01**	**<0.01**	0.271	**2.96**	**<0.01**	Chondrocyte and osteoblast determination and differentiation	(Komori, 2011; Zhang, *et al*, 2008)
S100B	bd			bd			bd			Inhibition of terminal differentiation of chondrocytes	(Saito, *et al*, 2007)
SHH	0.0008	4.29	0.37	0.0048	3.55	0.12	0.0028	0.73	0.23	Sonic hedgehog, Morphogen	(Currie and Ingham, 1996; Herzog, *et al*, 2003; Lewis and Eisen, 2001)
SMO	0.0427	**5.56**	**<0.01**	0.0951	**8.34**	**0.01**	0.0766	1.32	0.21	Hedgehog receptor	(Alcedo and Noll, 1997; Chen and Struhl, 1996)
SNAI2 (Slug)	0.018	2.15	0.09	0.025	0.75	0.29	0.029	0.61	0.06	Neural crest formation	(Aybar, *et al*, 2003; del Barrio and Nieto, 2002)
SNAI1 (Snail)	bd			bd			bd			Neural crest formation	(Aybar, Nieto and Mayor, 2003; del Barrio and Nieto, 2002)
SOX5	0.010	**0.44**	**<0.01**	0.002	**0.25**	**0.04**	0.007	0.81	0.15	Neural crest formation and chondrocyte differentiation	(Morales, *et al*, 2007; Perez-Alcala, *et al*, 2004; Zhang, Kong, Carlson, *et al*, 2008)
SOX6	0.008	1.05	0.46	0.003	0.41	0.11	0.004	1.29	0.19	Cartilage formation	(Ikeda, *et al*, 2005; Smits, *et al*, 2004; Smits and Lefebvre, 2003)
SOX9	0.055	3.80	0.09	0.002	0.35	0.11	0.006	0.65	0.12	Cartilage formation	(Bi, *et al*, 1999)
SOX10	0.001	0.84	0.43	0.004	1.02	0.12	0.010	0.60	0.09	Neural crest formation	(Kelsh, 2006)
SPAG6	bd			bd			bd			Sox5 downstream signal	(Kiselak, *et al*, 2010)
TWIST	bd			bd			bd			Melanocyte development	(Hoek, *et al*, 2004)
VCAN	0.026	1.75	0.15	0.201	0.69	0.201	0.038	1.78	0.17	Versican, chondrogenesis	(Shinomura, *et al*, 1993)

a) Relative qRT-PCR amplification level related to the level of ß-actin
bd; below detection. Back-ground levels were set to detection level
E; Embryonic day,
FD; fold difference = single-comb/Pea-comb expression levels
sc; single-comb
Statistical test: ANOVA *p*-value.

References

Alcedo, J. and Noll, M. (1997) Hedgehog and its patched-smoothened receptor complex: a novel signalling mechanism at the cell surface. *Biol Chem*, **378**, 583-590.

Aybar, M. J., Nieto, M. A. and Mayor, R. (2003) Snail precedes slug in the genetic cascade required for the specification and migration of the Xenopus neural crest. *Development*, **130**, 483-494.

Bi, W., Deng, J. M., Zhang, Z., Behringer, R. R. and de Crombrugghe, B. (1999) Sox9 is required for cartilage formation. *Nat Genet*, **22**, 85-89.
Boissy, R. E. and Nordlund, J. J. (1997) Molecular basis of congenital hypopigmentary disorders in humans: a review. *Pigment Cell Res*, **10**, 12-24.
Cheah, K. S., Stoker, N. G., Griffin, J. R., Grosveld, F. G. and Solomon, E. (1985) Identification and characterization of the human type II collagen gene (COL2A1). *Proc Natl Acad Sci U S A*, **82**, 2555-2559.
Chen, Y. and Struhl, G. (1996) Dual roles for patched in sequestering and transducing Hedgehog. *Cell*, **87**, 553-563.
Conway, S. J., Henderson, D. J. and Copp, A. J. (1997) Pax3 is required for cardiac neural crest migration in the mouse: evidence from the splotch (Sp2H) mutant. *Development*, **124**, 505-514.
Currie, P. D. and Ingham, P. W. (1996) Induction of a specific muscle cell type by a hedgehog-like protein in zebrafish. *Nature*, **382**, 452-455.
Dai, P., Akimaru, H., Tanaka, Y., Maekawa, T., Nakafuku, M. and Ishii, S. (1999) Sonic Hedgehog-induced activation of the Gli1 promoter is mediated by GLI3. *J Biol Chem*, **274**, 8143-8152.
del Barrio, M. G. and Nieto, M. A. (2002) Overexpression of Snail family members highlights their ability to promote chick neural crest formation. *Development*, **129**, 1583-1593.
Fafeur, V., Tulasne, D., Queva, C., Vercamer, C., Dimster, V., Mattot, V., Stehelin, D., Desbiens, X. and Vandenbunder, B. (1997) The ETS1 transcription factor is expressed during epithelial-mesenchymal transitions in the chick embryo and is activated in scatter factor-stimulated MDCK epithelial cells. *Cell Growth Differ*, **8**, 655-665.
Fichter, M., Korner, U., Schomburg, J., Jennings, L., Cole, A. A. and Mollenhauer, J. (2006) Collagen degradation products modulate matrix metalloproteinase expression in cultured articular chondrocytes. *J Orthop Res*, **24**, 63-70.
Gao, Z., Kim, G. H., Mackinnon, A. C., Flagg, A. E., Bassett, B., Earley, J. U. and Svensson, E. C. (2010) Ets1 is required for proper migration and differentiation of the cardiac neural crest. *Development*, **137**, 1543-1551.
Hartmann, C. and Tabin, C. J. (2000) Dual roles of Wnt signaling during chondrogenesis in the chicken limb. *Development*, **127**, 3141-3159.
Herzog, W., Zeng, X., Lele, Z., Sonntag, C., Ting, J. W., Chang, C. Y. and Hammerschmidt, M. (2003) Adenohypophysis formation in the zebrafish and its dependence on sonic hedgehog. *Dev Biol*, **254**, 36-49.
Hoek, K., Rimm, D. L., Williams, K. R., Zhao, H., Ariyan, S., Lin, A., Kluger, H. M., Berger, A. J., Cheng, E., Trombetta, E. S.*, et al* (2004) Expression profiling reveals novel pathways in the transformation of melanocytes to melanomas. *Cancer Res*, **64**, 5270-5282.
Ikeda, T., Kawaguchi, H., Kamekura, S., Ogata, N., Mori, Y., Nakamura, K., Ikegawa, S. and Chung, U. I. (2005) Distinct roles of Sox5, Sox6, and Sox9 in different stages of chondrogenic differentiation. *J Bone Miner Metab*, **23**, 337-340.
Itano, N., Sawai, T., Yoshida, M., Lenas, P., Yamada, Y., Imagawa, M., Shinomura, T., Hamaguchi, M., Yoshida, Y., Ohnuki, Y.*, et al* (1999) Three isoforms of mammalian hyaluronan synthases have distinct enzymatic properties. *Journal of Biological Chemistry*, **274**, 25085-25092.
Iwasaka, C., Tanaka, K., Abe, M. and Sato, Y. (1996) Ets-1 regulates angiogenesis by inducing the expression of urokinase-type plasminogen activator and matrix metalloproteinase-1 and the migration of vascular endothelial cells. *J Cell Physiol*, **169**, 522-531.
Kelsh, R. N. (2006) Sorting out Sox10 functions in neural crest development. *Bioessays*, **28**, 788-798.
Kiselak, E. A., Shen, X., Song, J., Gude, D. R., Wang, J., Brody, S. L., Strauss, J. F., 3rd and Zhang, Z. (2010) Transcriptional regulation of an axonemal central apparatus gene, sperm-associated antigen 6, by a SRY-related high mobility group transcription factor, S-SOX5. *J Biol Chem*, **285**, 30496-30505.
Klatt, A. R., Paul-Klausch, B., Klinger, G., Kuhn, G., Renno, J. H., Banerjee, M., Malchau, G. and Wielckens, K. (2009) A critical role for collagen II in cartilage matrix degradation: collagen II induces pro-inflammatory cytokines and MMPs in primary human chondrocytes. *J Orthop Res*, **27**, 65-70.

Kola, I., Brookes, S., Green, A. R., Garber, R., Tymms, M., Papas, T. S. and Seth, A. (1993) The Ets1 transcription factor is widely expressed during murine embryo development and is associated with mesodermal cells involved in morphogenetic processes such as organ formation. *Proc Natl Acad Sci U S A*, **90**, 7588-7592.

Komori, T. (2011) Signaling networks in RUNX2-dependent bone development. *J Cell Biochem*, **112**, 750-755.

Kwan, K. Y., Lam, M. M., Krsnik, Z., Kawasawa, Y. I., Lefebvre, V. and Sestan, N. (2008) SOX5 postmitotically regulates migration, postmigratory differentiation, and projections of subplate and deep-layer neocortical neurons. *Proc Natl Acad Sci U S A*, **105**, 16021-16026.

Lei, Y., Huang, K., Gao, C., Lau, Q. C., Pan, H., Xie, K., Li, J., Liu, R., Zhang, T., Xie, N.*, et al* (2011) Proteomics identification of ITGB3 as a key regulator in reactive oxygen species-induced migration and invasion of colorectal cancer cells. *Mol Cell Proteomics*, **10**, M110 005397.

Lewis, K. E. and Eisen, J. S. (2001) Hedgehog signaling is required for primary motoneuron induction in zebrafish. *Development*, **128**, 3485-3495.

Mankoo, B. S., Skuntz, S., Harrigan, I., Grigorieva, E., Candia, A., Wright, C. V., Arnheiter, H. and Pachnis, V. (2003) The concerted action of Meox homeobox genes is required upstream of genetic pathways essential for the formation, patterning and differentiation of somites. *Development*, **130**, 4655-4664.

Marigo, V., Johnson, R. L., Vortkamp, A. and Tabin, C. J. (1996) Sonic hedgehog differentially regulates expression of GLI and GLI3 during limb development. *Dev Biol*, **180**, 273-283.

Merlino, G. T., McKeon, C., de Crombrugghe, B. and Pastan, I. (1983) Regulation of the expression of genes encoding types I, II, and III collagen during chick embryonic development. *J Biol Chem*, **258**, 10041-10048.

Monsoro-Burq, A. H., Duprez, D., Watanabe, Y., Bontoux, M., Vincent, C., Brickell, P. and Le Douarin, N. (1996) The role of bone morphogenetic proteins in vertebral development. *Development*, **122**, 3607-3616.

Morales, A. V., Perez-Alcala, S. and Barbas, J. A. (2007) Dynamic Sox5 protein expression during cranial ganglia development. *Dev Dyn*, **236**, 2702-2707.

Nakata, K., Nakahara, H., Kimura, T., Kojima, A., Iwasaki, M., Caplan, A. I. and Ono, K. (1992) Collagen gene expression during chondrogenesis from chick periosteum-derived cells. *FEBS Lett*, **299**, 278-282.

Oda, N., Abe, M. and Sato, Y. (1999) ETS-1 converts endothelial cells to the angiogenic phenotype by inducing the expression of matrix metalloproteinases and integrin beta3. *J Cell Physiol*, **178**, 121-132.

Opdecamp, K., Nakayama, A., Nguyen, M. T., Hodgkinson, C. A., Pavan, W. J. and Arnheiter, H. (1997) Melanocyte development in vivo and in neural crest cell cultures: crucial dependence on the Mitf basic-helix-loop-helix-zipper transcription factor. *Development*, **124**, 2377-2386.

Perez-Alcala, S., Nieto, M. A. and Barbas, J. A. (2004) LSox5 regulates RhoB expression in the neural tube and promotes generation of the neural crest. *Development*, **131**, 4455-4465.

Pla, P., Solov'eva, O., Moore, R., Alberti, C., Kunisada, T. and Larue, L. (2004) Dct::lacZ ES cells: a novel cellular model to study melanocyte determination and differentiation. *Pigment Cell Res*, **17**, 142-149.

Pourquie, O., Fan, C. M., Coltey, M., Hirsinger, E., Watanabe, Y., Breant, C., Francis-West, P., Brickell, P., Tessier-Lavigne, M. and Le Douarin, N. M. (1996) Lateral and axial signals involved in avian somite patterning: a role for BMP4. *Cell*, **84**, 461-471.

Reijntjes, S., Stricker, S. and Mankoo, B. S. (2007) A comparative analysis of Meox1 and Meox2 in the developing somites and limbs of the chick embryo. *Int J Dev Biol*, **51**, 753-759.

Rescan, P. Y. and Ralliere, C. (2010) A Sox5 gene is expressed in the myogenic lineage during trout embryonic development. *Int J Dev Biol*, **54**, 913-918.

Retief, E., Parker, M. I. and Retief, A. E. (1985) Regional chromosome mapping of human collagen genes alpha 2(I) and alpha 1(I) (COLIA2 and COLIA1). *Hum Genet*, **69**, 304-308.

Rothhammer, T., Hahne, J. C., Florin, A., Poser, I., Soncin, F., Wernert, N. and Bosserhoff, A. K. (2004) The Ets-1 transcription factor is involved in the development and invasion of malignant melanoma. *Cell Mol Life Sci*, **61**, 118-128.

Saito, T., Ikeda, T., Nakamura, K., Chung, U. I. and Kawaguchi, H. (2007) S100A1 and S100B, transcriptional targets of SOX trio, inhibit terminal differentiation of chondrocytes. *EMBO Rep*, **8**, 504-509.

Shinomura, T., Nishida, Y., Ito, K. and Kimata, K. (1993) cDNA cloning of PG-M, a large chondroitin sulfate proteoglycan expressed during chondrogenesis in chick limb buds. Alternative spliced multiforms of PG-M and their relationships to versican. *J Biol Chem*, **268**, 14461-14469.

Smits, P., Dy, P., Mitra, S. and Lefebvre, V. (2004) Sox5 and Sox6 are needed to develop and maintain source, columnar, and hypertrophic chondrocytes in the cartilage growth plate. *J Cell Biol*, **164**, 747-758.

Smits, P. and Lefebvre, V. (2003) Sox5 and Sox6 are required for notochord extracellular matrix sheath formation, notochord cell survival and development of the nucleus pulposus of intervertebral discs. *Development*, **130**, 1135-1148.

Spicer, A. P., Olson, J. S. and McDonald, J. A. (1997a) Molecular cloning and characterization of a cDNA encoding the third putative mammalian hyaluronan synthase. *J Biol Chem*, **272**, 8957-8961.

Spicer, A. P., Seldin, M. F., Olsen, A. S., Brown, N., Wells, D. E., Doggett, N. A., Itano, N., Kimata, K., Inazawa, J. and McDonald, J. A. (1997b) Chromosomal localization of the human and mouse hyaluronan synthase genes. *Genomics*, **41**, 493-497.

Thomas, A. J. and Erickson, C. A. (2008) The making of a melanocyte: the specification of melanoblasts from the neural crest. *Pigment Cell Melanoma Res*, **21**, 598-610.

Tucker, R. P. (2004) Neural crest cells: a model for invasive behavior. *Int J Biochem Cell Biol*, **36**, 173-177.

Vortkamp, A., Lee, K., Lanske, B., Segre, G. V., Kronenberg, H. M. and Tabin, C. J. (1996) Regulation of rate of cartilage differentiation by Indian hedgehog and PTH-related protein. *Science*, **273**, 613-622.

Wakabayashi, T., Matsumine, A., Nakazora, S., Hasegawa, M., Iino, T., Ota, H., Sonoda, H., Sudo, A. and Uchida, A. (2010) Fibulin-3 negatively regulates chondrocyte differentiation. *Biochem Biophys Res Commun*, **391**, 1116-1121.

Woods, A. and Beier, F. (2006) RhoA/ROCK signaling regulates chondrogenesis in a context-dependent manner. *J Biol Chem*, **281**, 13134-13140.

Ying, Z., Jin, L., Palmer, T. and Webb, R. C. (2006) Angiotensin II up-regulates the leukemia-associated Rho guanine nucleotide exchange factor (RhoGEF), a regulator of G protein signaling domain-containing RhoGEF, in vascular smooth muscle cells. *Mol Pharmacol*, **69**, 932-940.

Yu, H., Smallwood, P. M., Wang, Y., Vidaltamayo, R., Reed, R. and Nathans, J. (2010) Frizzled 1 and frizzled 2 genes function in palate, ventricular septum and neural tube closure: general implications for tissue fusion processes. *Development*, **137**, 3707-3717.

Zhang, Y., Kong, L., Carlson, C. S. and Liu, C. J. (2008) Cbfa1-dependent expression of an interferon-inducible p204 protein is required for chondrocyte differentiation. *Cell Death Differ*, **15**, 1760-1771.
